# Outer membrane vesicles-transmitted virulence genes mediate the emergence of new antimicrobial-resistant hypervirulent *Klebsiella pneumoniae*

**DOI:** 10.1080/22221751.2022.2065935

**Published:** 2022-05-23

**Authors:** Yuneng Hua, Jingyu Wang, Mei Huang, Yiyi Huang, Ruyi Zhang, Fan Bu, Biao Yang, Juanjiang Chen, Xiaomin Lin, Xiumei Hu, Lei Zheng, Qian Wang

**Affiliations:** a Center for Clinical Laboratory, Zhujiang Hospital, Southern Medical University, Guangzhou, People’s Republic of China; b Department of Laboratory Medicine, Nanfang Hospital, Southern Medical University, Guangzhou, People’s Republic of China

**Keywords:** Outer membrane vesicles, hypervirulent *klebsiella pneumoniae*, horizontal gene transfer, virulence genes

## Abstract

Hypervirulent *Klebsiella pneumoniae* (hvKp) is a notorious clinical pathogen that is more likely to cause severe primary and metastatic abscesses. The dissemination of antimicrobial-resistant hvKp isolates has been reported worldwide, posing a great challenge and severe clinical threat. However, the mechanisms of antimicrobial-resistant hvKp isolates prevalent worldwide are not well precise. Outer membrane vesicles (OMVs) secreted from gram-negative bacteria are an important vehicle for delivering effector molecules inter- and intra-species. To explore whether OMVs as the vector of virulence genes horizontal transfer among *Klebsiella pneumoniae* and to explain the potential mechanism for the development of antimicrobial-resistant hvKp isolates, we isolated OMVs from hvKp and classical *Klebsiella pneumoniae* (cKp) by sequential differential centrifugation, respectively. Then, the characteristics and contents of hvKp-OMVs and cKp-OMVs were analyzed. These hvKp-OMVs contain virulence genes, which could be transferred from hvKp horizontally to extended-spectrum beta lactamase (ESBL)-producing cKp, leading to the production of antimicrobial-resistant hypervirulent transformants. Further experiments confirmed the transformants exhibited antimicrobial resistance and hypervirulent phenotypes *in vitro* and *in vivo*. In short, this work demonstrated that hvKp-OMVs facilitated virulence genes transfer, allowing an increase in the virulence level of ESBL-producing cKp and providing a new mechanism for the emergence of antimicrobial-resistant hvKp isolates.

## Introduction

Hypervirulent *Klebsiella pneumoniae* (hvKp) is a notorious clinical pathogen with a hypermucoviscosity phenotype, more likely to cause primary and metastatic abscesses, clinically characterized by organ or life-threatening infections in community-dwelling healthy hosts [1,2]. hvKp contains a large virulence plasmid that harbours many virulence-encoding genes, considered genetic factors that confer hvKp’s hypervirulent phenotype [3,4]. These genetic determinants of hypervirulence display phenotypes associated with increased capsule production, aerobactin production and mucoviscosity [5]. The continuous evolution of *Klebsiella pneumoniae* (KP) depends on acquiring resistance-encoding and hypervirulence-encoding mobile genetic elements [6]. For a long period, hvKp strains were rarely resistant to commonly used antimicrobials due to some limitation for gene acquisition. The significantly thickened capsules’ polysaccharide of hvKp as a constraint on horizontal gene transfer, and chromosomal recombination is rare in hvKp, with decreased plasmid diversity driving a reduction in pan-genome diversity. These factors lead to the fact that the clonal diversity of hvKp is much less than that of multidrug-resistant (MDR) counterparts, and the efficiency of MDR clones in acquiring virulence plasmids far exceeds the efficiency of hvKp clones in developing drug-resistant plasmids [7,8]. However, antimicrobial-resistant hvKp isolates have now been reported worldwide. The emergence of these strains may be because the classical *Klebsiella pneumoniae* (cKp) strains to acquire pLVPK-like virulence plasmid or the acquisition of resistance determinants elements by hvKp strains [9]. It is important to elucidate the underlying mechanisms that contribute to the spread of virulence elements that promote the emergence of antimicrobial-resistant hvKp.

Bacterial outer membrane vesicles (OMVs) conduct diverse functions, including the delivery of genetic materials or virulence factors, regulation of intercellular communication, acting as a decoy for bacterial toxins and modulation of host immune responses [10]. Almost all gram-negative bacteria ubiquitously produce and release OMVs that contain numerous functional lipids, proteins, RNAs and DNAs, which can be horizontally transferred to the recipient bacteria or host cells [11]. OMVs represent a unique bacterial secretion pathway that selects and protects its cargo, allowing bacteria to act on and interact with their environment over extended distances without the risk of direct contact [12]. As observed in previous studies, it is revealed that OMVs protect luminal genes or plasmids against DNases and function as an effective horizontal gene transfer system [13].

Importantly, in Federica et al., genetically engineered *Klebsiella pneumoniae*-OMVs serve as vectors for antimicrobial resistance genes spread in the microbial communities [14]. hvKp similarly secretes outer membrane vesicles lumen-contained various virulence factors and bioactive substances, stimulating the inflammatory response [15]. However, it is unclear whether these hvKp-derived OMVs promote the horizontal transfer of mobile virulent elements between bacteria and thus produce the antimicrobial-resistant hvKp isolates. Our work first reported that hvKp-OMVs can transfer virulence genes to extended spectrum beta lactamase (ESBL)-producing cKp, resulting in increased mucoviscosity and quantification of capsule, thus contributing to enhanced virulence levels of the latter. ESBL-producing hvKp isolates exhibit hypervirulent phenotype and simultaneously resistant to almost all beta-lactam groups of antibiotics except for carbapenems and cephamycins. In summary, our data reveal the essential role of hvKp-OMVs in the transmission of virulence genes among *Klebsiella pneumoniae* strains, elucidating the potential mechanisms for developing antimicrobial-resistant hvKp isolates.

## Results

### Characterization of OMVs derived from hvKp

**Figure 1.**
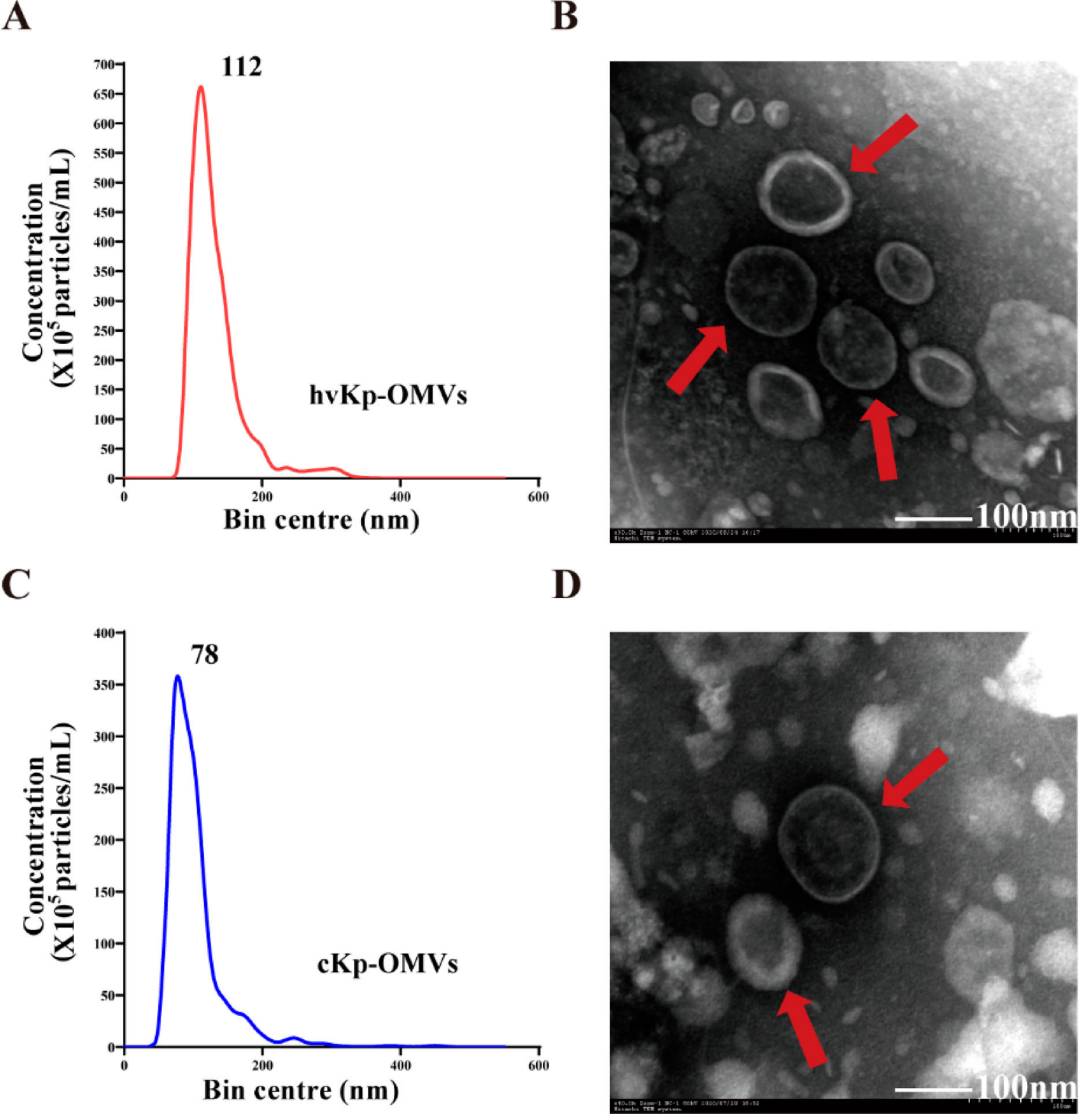
Purification and characterization of OMVs derived from hvKp and cKp strains. (A) NTA of hvKp-OMVs and (C) cKp-OMVs (*n* = 3). (B) TEM of hvKp-OMVs (arrows) and (D) cKp-OMVs (arrows), scale bar = 100 nm.

hvKp and cKp isolates were grown in Luria–Bertani (LB) broth, and the culture supernatants were collected after 6 h of incubation. hvKp-OMVs and cKp-OMVs were isolated from the harvested broth and characterized for morphology and size. A transmission electron microscope (TEM) showed that these purified OMVs were oval and spherical. There was no significant difference between hvKp-OMVs (Figure 1B) and cKp-OMVs (Figure 1D) in the morphology. Nanoparticle tracking analysis (NTA) showed that hvKp-OMVs were larger (54-634 nm, median size 112 nm) (Figure 1A) than cKp-OMVs (17–523 nm, median 78 nm), which was consistent with what was described previously (Figure 1C). The OMVs were well purified, no bacteria were observed under the microscope, and the contaminated control on the plates grew nothing.

### hvKp-OMVs are enriched in bacteria genetic elements and functional proteins

**Figure 2.**
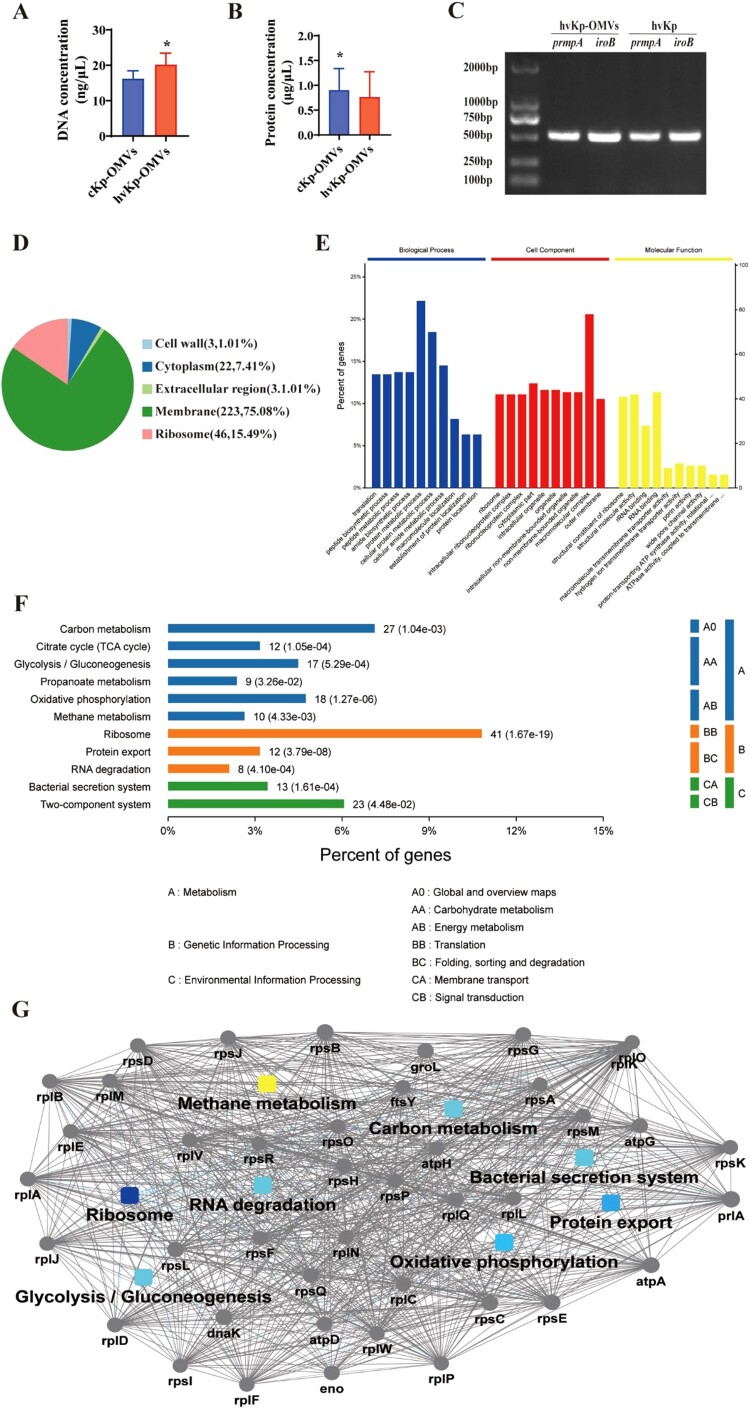
Protein and DNA concentration of the OMVs. (A) DNA concentration of hvKp-OMVs and cKp-OMVs (*n* = 3). (B) Protein concentration of hvKp-OMVs and cKp-OMVs (*n* = 3). (C) PCR profile of the hvKp-OMVs and hvKp strains used in this study. (D) Cellular localization of protein from hvKp-OMVs. (E) Biological Process (left), Cellular Component (middle) and Molecular Function (right) of protein from hvKp-OMVs. (F) Bioinformatics analyses of Kyoto Encyclopedia of Genes and Genomes (KEGG) and (G) Protein-Protein Interaction Networks (PPI) were performed on protein from hvKp-OMVs.

The mean DNA concentrations (average of the three experiments) of the hvKp-OMVs and cKp-OMVs were 21.17 and 17.56 ng/µL, respectively (Figure 2A). The mean protein concentrations (average of the three experiments) of the hvKp-OMVs and cKp-OMVs were 0.76 and 0.91 µg/µL, respectively (Figure 2B). As demonstrated in Figure 2(C), DNA purified from the OMVs gave specific amplified products for *iroB* and *prmpA*.

A total of 1177 proteins were detected by Nano LC-MS/MS analysis in hvKp-OMVs. Two hundred twenty-three membrane-associated proteins (75.08%), 46 ribosomal proteins (15.49%), 22 cytosolic proteins (7.41%), 3 cell wall proteins (1.01%) and 3 extracellular region proteins (1.01%) were found in the vesicles (Figure 2D). Vesicle proteins were categorized following subcellular localization site, biological function, and molecular function (Figure 2E). Further bioinformatics analyses of the Kyoto Encyclopedia of Genes and Genomes (KEGG) and Protein–Protein Interaction Networks (PPI) were performed, which is shown in (Figure 2F, G).

### hvKp-OMVs mediate virulence genes transfer to cKp strains

To investigate whether hvKp-OMVs mediated virulence genes transfer to cKp strains, we performed transformation experiments to confirm the potential of hvKp-OMVs to deliver virulence genes to ESBL-producing cKp. Exogenous DNA and protein were digested by DNase and proteinase K. Treated OMVs were used to transform ESBL-producing cKp. After 24 h, treated cells were spread onto an LB agar plate to identify successful transformants. Two strains of successful transformants, cKp-TC1 and cKp-TC2, were selected for further experiments.

**Figure 3.**
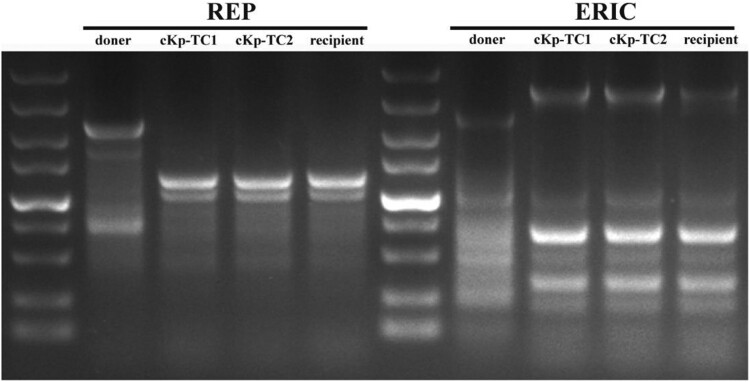
Agarose gel electrophoresis showed REP-PCR products with expected images. REP-PCR profile of the hvKp, cKp-TC1, cKp-TC2 and cKp strains used in this study. The primers used to perform repetitive extragenic palindromic PCR (REP-PCR) are REP (left) and ERIC (right), respectively.

Antibiotic sensitivity tests and REP-PCR assays were established on donor, recipient, and transformants. The results showed that these transformants exhibited minimal inhibitory concentration (MIC) and agarose gel electrophoresis images similar to those of the recipient strains. There was no significant difference in the band characteristics of the REP-PCR amplified fragments between the recipient strains and transformants (Figure 3). Therefore, the contamination of the donor strains was excluded. According to PCR results, the transformants contained a marker gene of the virulence plasmid (*prmpA*). Overall, these results supported the idea that these transformants had drug resistance-associated phenotype and hypervirulence-associated phenotype (Table 2). These data indicated that the hvKp-OMVs could effectively transmit the virulence genes to ESBL-producing cKp.

**Table 2.** Phenotypic and genotypic characteristics of donor, transformant, and recipient strains.

Strain	MIC(μg ml^−1^)							*prmpA*
	Cefazolin	Cefotaxime	Imipenem	Meropenem	Ampicillin	Aztreonam	Tetracycline	
cKp	16	2	2	<1	>16	>16	>8	–
cKp-TC1	16	2	2	<1	>16	>16	>8	+
cKp-TC2	16	2	2	<1	>16	>16	>8	+
hvKp	≤4	≤1	≤1	<1	16	≤2	≤2	+

### hvKp-OMVs enhance the virulence level of cKp strains

**Figure 4.**
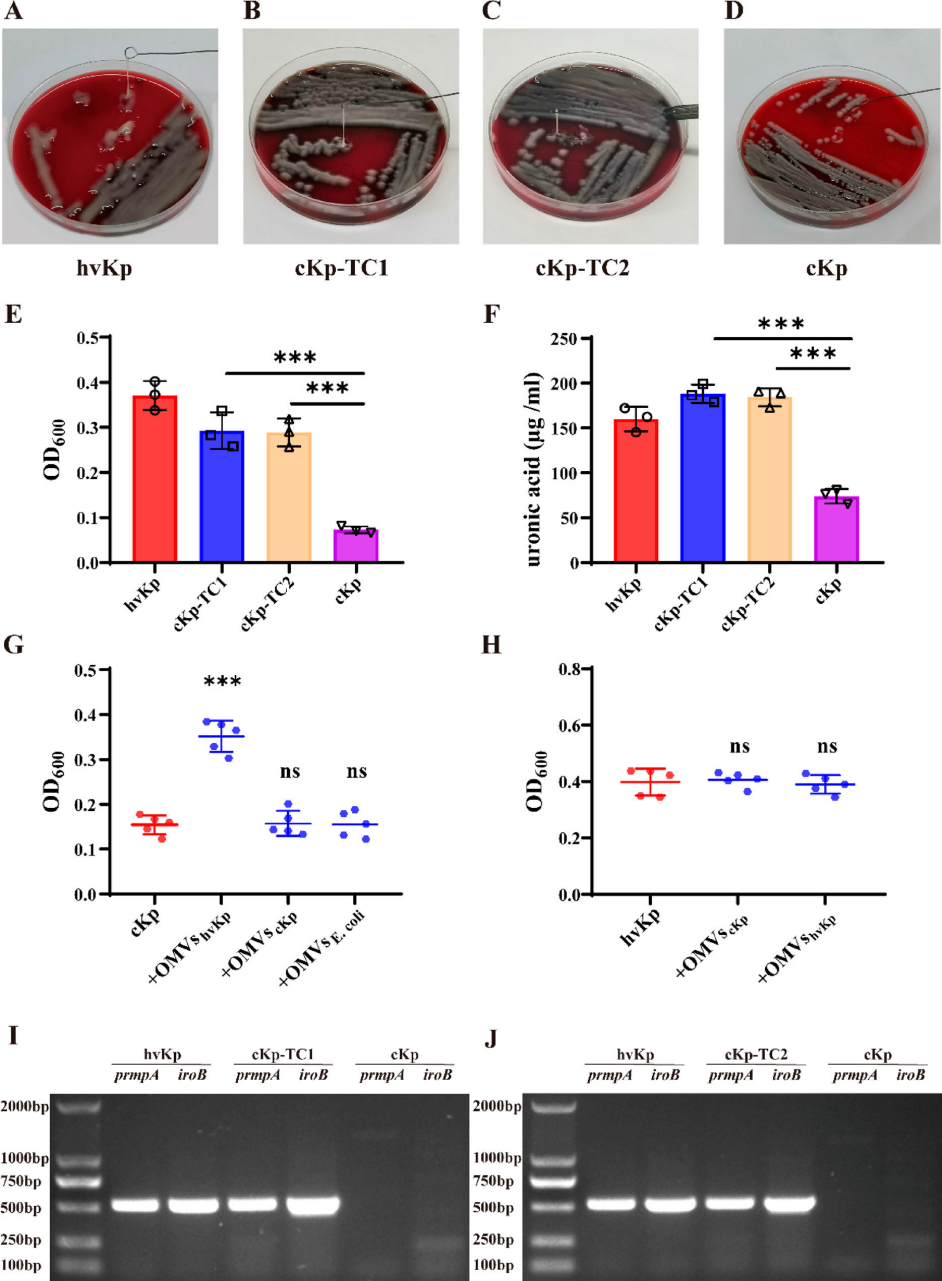
hvKp-OMVs enhanced mucoviscosity and capsule production of cKp strains. (A) String tests on blood agar plates of hvKp, (B) cKp-TC1, (C) cKp-TC2, (D) cKp strains. (E) Mucoviscosity and (F) uronic acid production of hvKp, cKp-TC1, cKp-TC2 and cKp strains (+OMVs_hvKp_-100 μg). Results are presented as mean ± SEM, ***P < 0.001. (G) hvKp-OMVs could induce an increased level of mucoviscosity in cKp strains (+OMVs_hvKp_-500 μg), while the level of mucoviscosity in cKp strains is not enhanced by E.coli-OMVs (+OMVs_E.coli_-500 μg) and cKp-OMVs (+OMVs_cKp_-500 μg). Wilcoxon paired *t*-test, ***P < 0.001, significant difference. Wilcoxon paired *t*-test, ns *P* > 0.05, no significant difference. (H) cKp-OMVs (+OMVs_cKp_-500 μg) could not induce the increased level of mucoviscosity in hvKp strains, while the level of mucoviscosity in hvKp strains is not enhanced by its own OMVs even at higher concentrations (+OMVs_hvKp_-500 μg). Wilcoxon paired *t*-test, ns *P* > 0.05, no significant difference. (I)(J) PCR amplified fragments of the hvKp, cKp-TC1, cKp-TC2 and cKp strains, respectively.

To verify whether the transformants acquired virulence genes, we conducted hypermucoviscosity assay to test for successful transformation. Virulence determinants carried by the pLVPK-like plasmid is associated with hypervirulent phenotype, including regulator of the mucoid phenotype (*prmpA/A2*), salmochelin (*iroBCDN*) and aerobactin (*iucABCD-iutA*). String tests on blood agar plates showed a marked increase in length by stretching colonies of the transformants compared with the recipient strains (Figure 4A, B, C, and D). The results established that incubation of hvKp-OMVs with the ESBL-producing cKp strains could increase the level of mucoviscosity and capsular polysaccharide (uronic acid) in most of the transformants, which was closely related to the virulence expression of hyper-virulent strains (Figure 4E, F, +OMVs_hvKp_-100 µg). In our work, we found that hvKp-OMVs at least 100 µg can cause an increased level of mucoviscosity of ESBL-producing cKp.

Only the OMVs secreted by hvKp could induce the increased level of mucoviscosity and capsular polysaccharide (uronic acid) in the ESBL-producing cKp strains, as OMVs isolated from cKp or E.coli strains could not increase the level of mucoviscosity in the ESBL-producing cKp strains (Figure 4G, +OMVs_cKp_-500μg, +OMVs_E.coli_-500μg). Furthermore, cKp-OMVs could not cause increased mucoviscosity of the hype-virulent strains (Figure 4H, +OMVs_cKp_-500 µg), and hvKp strains acquisition of its OMVs could not enhance the level of mucoviscosity even at a higher concentration (Figure 4H, +OMVs_hvKp_-500 µg). The presence of plasmid-borne genes *prmpA* and *iroB* as a marker of the virulence plasmid in the transformants was determined by PCR (Figure 4I, J).

### The virulence level of transformants was tested by the mouse lethality assay

**Figure 5.**
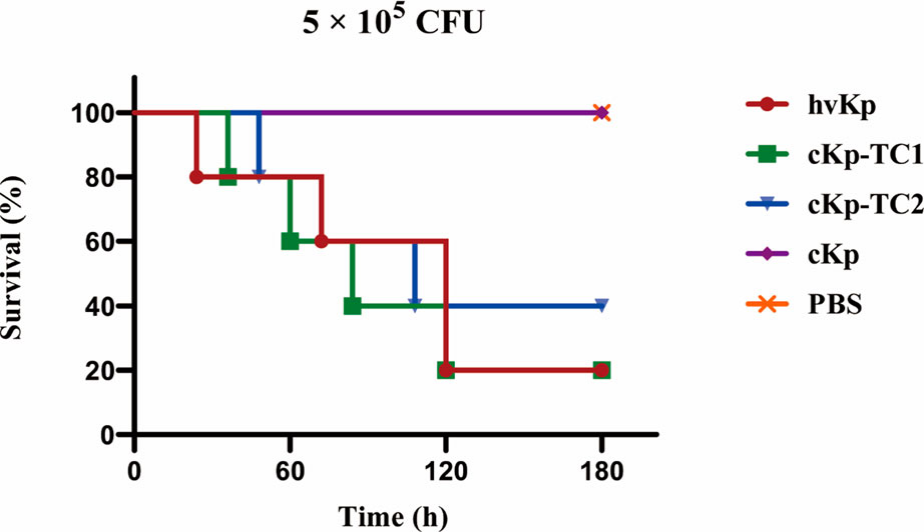
Virulence potential of hvKp, cKp-TC1, cKp-TC2 and cKp strains with an inoculum of 5 × 10^5^ CFU at 120 h after infection in a mouse lethality assay (*n* = 5). The log-rank Mantel–Cox test was used for the analysis of survival curves.

The virulence level of these two transformants was further detected by the mouse lethality assay. At 5.0 × 10^5^ colony-forming units (CFU) of bacteria in infected mice, cKp-TC1 and cKp-TC2 resulted in 80% and 60% mortality at 120 h, respectively, a level slightly lower than that of hvKp but substantially higher than that of ESBL-producing cKp. Furthermore, when infected with the same dose of ESBL-producing cKp or phosphate buffer saline (PBS), the post-infection mortality rate was 0% at 120 h (Figure 5). These data confirm that the acquisition of virulence plasmids by ESBL-producing cKp strains resulted in an enhanced level of virulence comparable to that of the hvKp strains, as demonstrated by the ability to cause a higher mortality rate in the mouse lethality assay.

### Transformants exhibited stable hypermucoviscosity phenotype

**Figure 6.**
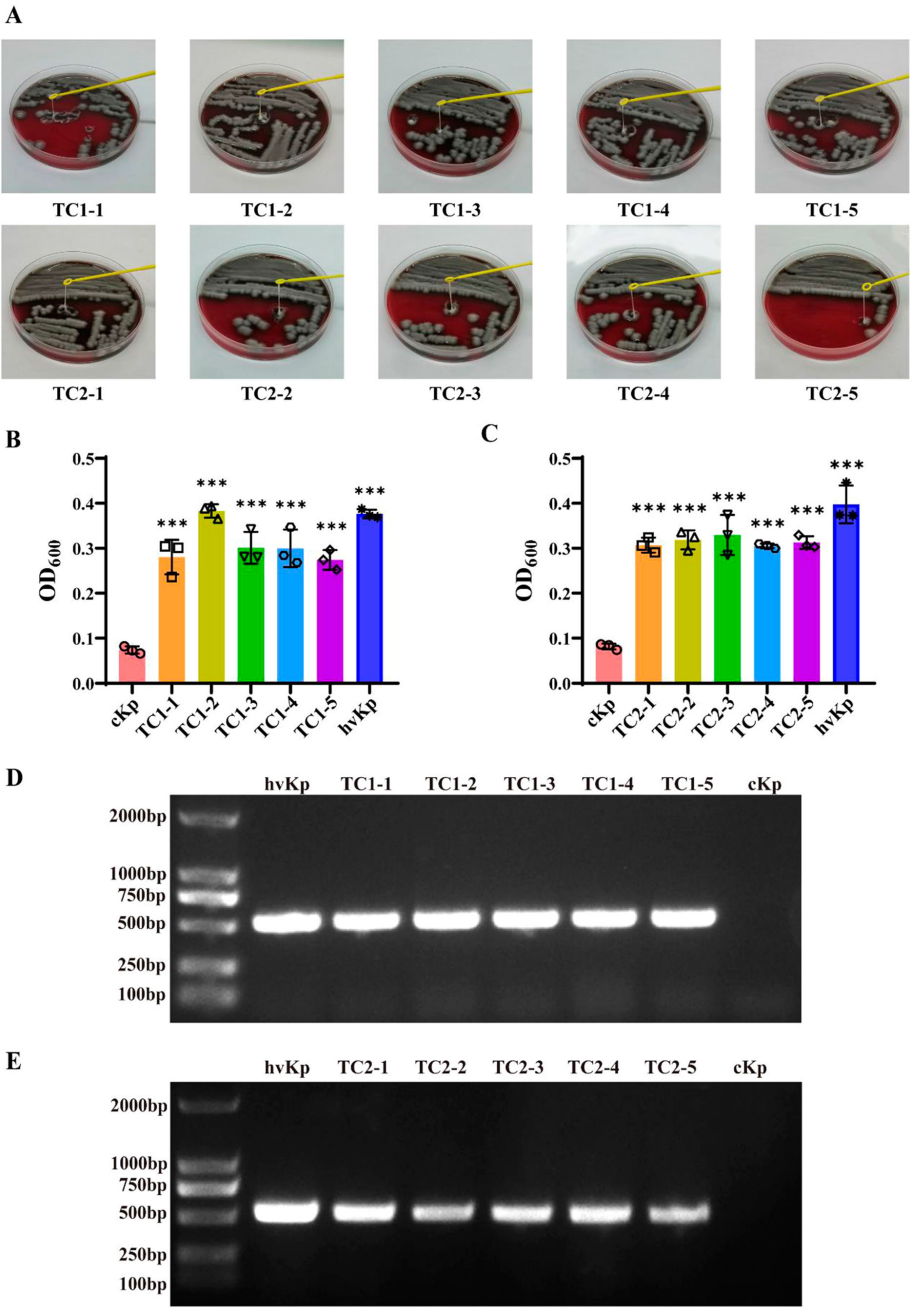
Transformants exhibited stable hypermucoviscosity phenotype after several generations. hvKp-OMVs enhanced mucoviscosity of cKp strains. (A) String tests on blood agar plates of five generations of cKp-TC1 and cKp-TC2. (B)Mucoviscosity of five generations of cKp-TC1 and (C)cKp-TC2. (+OMV_ShvKp_-100 μg). Results are presented as mean ± SEM, ***P <0.001. (D) PCR amplified fragments of five generations of cKp-TC1 and (E) cKp-TC2 (*prmpA*).

To determine if the transformants exhibited stable hypermucoviscosity phenotype, all transformants were passaged for five generations, and then the expression of the hyper-mucoviscosity phenotype of these five generations was measured. String tests on blood agar plates showed a marked increase in length by stretching these strains (Figure 6A). The results demonstrated that the level of mucoviscosity was significantly increased in these five generations and the expression level of hypermucoviscosity phenotype without the impact from the passage. (Figure 6B, C)

Plasmid-borne gene *prmpA* of these five generations were analyzed using PCR assay, showing that the transformants' acquisition of virulence genes still contained *prmpA* after several generations (Figure 6D, E).

## Discussion

*Klebsiella pneumoniae*, a concerning global pathogen, is widely recognized as a cause of hospital- and community-acquired infections posing a mounting threat. With its evolution, it has gradually evolved into classical *Klebsiella pneumoniae* (cKp) and hypervirulent *Klebsiella pneumoniae* (hvKp) [16]. However, hvKp is more virulent than cKp, and can cause nosocomial and community-acquired infections in healthy individuals [3]. cKp often carries antimicrobial-resistant genetic elements, such as β-lactamase and carbapenem genes. Unlike cKp, hvKp harbours pLVPK-like virulence plasmids encoding important virulence factors such as capsular polysaccharides regulator (*prmpA/A2*) and siderophores (*iroBCD/iucABCD*). hvKp strains acquire antimicrobial-resistance genetic elements or drug-resistant cKp strains obtain pLVPK-like virulence plasmids, leading to the emergence of multidrug-resistant hvKp isolates. However, at least two reasons limit the emergence of those strains. One thought is that pLVPK-like virulence plasmids are non-conjugative. In addition, hvKp has some sort of genetic limitation and the constraint of significantly thickening the capsule, making it challenging to obtain mobile genetic elements, which is one of the prominent features of hvKp [7]. The virulent plasmid, once confined to hvKp, has gradually spread to cKp [17]. The emergence and dissemination of multidrug-resistant hvKp isolates undoubtedly pose an alarming challenge to antimicrobial therapy and public health [18].

Outer membrane vesicles (OMVs) are nano-sized vesicles with various biological properties secreted by various gram-negative bacteria under physiological and pathological conditions [19]. OMVs play a critical role in cell–cell communication, directing the contents to be nearby or distant cells or tissues and assisting cells in exchanging proteins, lipids, nucleic acids and virulence factors [20]. Work described OMVs as a carrier for horizontal gene transfer (HGT) is known about *A. baumannii* OMVs facilitate intra- or inter-species transfer of plasmids containing OXA-24 Carbapenemase Gene and *blaNDM-1* gene to surrounding bacterial [21,22]. Moreover, recent studies demonstrate that OMVs mediate the transfer of virulence genes from *Escherichia coli* O157:H7 to other enteric bacteria [23].

In this work, the OMVs were extracted from hvKp and cKp, respectively. The results of TEM and NTA showed that OMVs have typical structures. There was no significant difference in the morphology of hvKp-OMVs and cKp-OMVs, but the particle size of hvKp-OMVs was larger than that of cKp-OMVs. This particle size difference may be due to the significantly thickened capsule of hvKp isolates. According to previous studies [24], the size of vesicles of capsulated bacterial or fungal strains was generally larger than that of non-capsulated strains, and the specific mechanism remained to be clarified. The mass spectrometric results suggested that a total of 1177 proteins in hvKp-OMVs, including outer and inner membrane proteins.

Recent work confirmed that *Klebsiella pneumoniae*-OMVs could carry drug-resistant genetic elements for horizontal gene transfer (HGT) between bacteria, and the DNA wrapped in them was not affected by DNases, giving this new HGT mechanism an additional advantage [21]. We proved that hvKp could release OMVs containing the vital virulence genes, including the regulator of mucoid phenotype (*prmpA*) and siderophores (*iroB*). After hvKp-OMVs carried virulence genes were transferred to ESBL-producing cKp, the transformants showed enhanced hypermucoviscosity phenotype. Key genes related to the virulent plasmids were detectable by PCR, such as *iroB*, *prmpA*, and *iutA*. When the hvKp, ESBL-producing cKp, and the transformants were injected intravenously into mice, the mouse survival rate of the transformants was significantly decreased compared with that of the ESBL-producing cKp strains, closer to the high virulence level, proving that the transformants displayed hypervirulent and multidrug-resistant phenotypes.

To verify our work was not only applied to a specific strain, but OMVs-mediated transformation experiments were conducted on new *Klebsiella pneumoniae* clinical isolates. PCR-based assays were performed on *peg-344, iroB, iucA, prmpA* and *prmpA2* genes. Results suggested that *iroB, iucA, prmpA* and *prmpA2* genes of this clinical isolates are positive (Supplementary Fig 2). Two strains of successful transformants, cKp-TC3 and cKp-TC4, were selected for further experiments. The viscous string showed a marked increase in length by stretching bacterial colonies of the transformants compared with the recipient strains on blood agar plates (Supplementary Fig 3A). OMVs-mediated transformation experiments demonstrated that OMVs derived from this new hypervirulent *Klebsiella pneumoniae* clinical isolates enhanced the level of the mucoviscosity of cKp strains. (Supplementary Fig3B,+OMVs_hvKp_-100 µg). *prmpA* and *iroB* genes as a molecular marker of the virulence plasmid in the successful transformants were determined by the PCR assay (Supplementary Fig 3C).

Antimicrobial-resistant hvKp infections have been widely disseminated during the past years, which exhibit severe challenges to the prevention and treatment of these strains. The main limitation of this study is that the molecular mechanism underlying the virulence genes transfer event, which enables the emergence of antimicrobial-resistant hvKp isolates is not completely clear. Further explorations are required to find out the specific mechanism of DNA wrapping into the vesicles and to clarify how the vesicles enter the recipient bacterial strains. In addition, although cKp-OMVs could not cause increased mucoviscosity of the hype-virulent strains, and hvKp strains acquisition of its own OMVs could not enhance the level of mucoviscosity even though it is possible at higher concentration, the potential roles of cKp-OMVs in mediating resistance-encoding mobile genetic elements transfer to hvKp strains remain unclear. Similarly, we wonder whether drug-resistant hvKp simultaneously disseminates drug-resistance and hypervirulence genetic factors. The role of OMVs from the drug-resistant hvKp isolates in delivering mobile genetic elements among *Klebsiella pneumoniae* deserves further exploration.

In summary, we demonstrated for the first time that the hvKp-OMVs could carry key virulence genes, leading to the increased level of mucoviscosity and virulence expression in drug-resistant strains’ acquisition of virulence determinants. First this work elucidates a new mechanism governing the emergence and dissemination of antimicrobial-resistant hypervirulent *klebsiella pneumoniae*.

## Materials and methods

### Klebsiella strain

A *Klebsiella pneumoniae* clinical isolate were detected in the pharyngeal swab of a 63-year-old man in an otorhinolaryngology clinic of Nanfang Hospital in Guangdong Province in 2019, which was identified by VITEK MS system (BioMérieux, China) or BD Phoenix 100 automatic identification and susceptibility testing system (Becton Dickinson, USA).The patient was referred to an otorhinolaryngology clinic due to parotid abscess.

### Whole Genome sequencing

Genomic DNA was extracted from *K. pneumoniae* clinical isolates using the bacterial genomic DNA extraction kit (TIANGEN Biotech, China) and sequenced by the MinION (Oxford Nanopore Technologies) platform. The strain had a 5.45-Mb chromosome and four main plasmids with sizes of 177,011base pairs (bp), 113,492 (bp), 10,455 (bp) and 9,319 (bp), respectively (Supplementary Table 1). The whole sequence of *K. pneumoniae* clinical isolates had been submitted to the GenBank databases (accession number JAKFHZ000000000).

### String test

The strain was then inoculated onto a blood agar plate and showed a positive string test, in which the viscous string was greater than 5 mm in length when stretching bacterial colonies grown overnight at 37°C using a bacteriology inoculation loop on an agar plate, indicating that it showed hypermucoviscosity phenotype.

### Polymerase chain reaction

The extraction of genomic DNA from all *K. pneumoniae* isolates was performed with a bacterial genomic DNA extraction kit (TIANGEN Biotech, China). PCR-based assays were performed on *peg-344, iroB, iucA, prmpA* and *prmpA2* genes of these strains for the presence of virulence-encoding plasmid, as previously reported (Supplementary Figure 1) [25]. Not all hvKp are hypermucoviscous and the detection of five virulence genes markers (*peg-344, iroB, iucA, prmpA, and prmpA2*) provides >95% diagnostic accuracy for distinguishing hvKp from cKp. The five markers were detected, and the positive rate of those ≥2 combined with the clinical features was preliminarily determined to be a hypervirulent *Klebsiella pneumoniae.* Table 1 lists the specific primers, PCR conditions and product size.

**Table 1.** Oligonucleotides used in this work.

Primer	Sequence (5′ to 3′)	Length (bp)	Use(s)
*prmpA* Forw	ACTGGGCTACCTCTGCTTCA	536	PCR
*prmpA* Rev	CTTGCATGAGCCATCTTTCA		
*iroB* Forw	CCCTGGCATATCAAAGGCGT	534	PCR
*iroB* Rev	GACAACAACGCGGGCATTTA		
*peg-344* Forw	TGGGGTTATTCTTTCGCT	508	PCR
*peg-344* Rev	TTTCCAAGCTTACTGCAATT		
*prmpA2* Forw	GTGCAATAAGGATGTTACATTA	430	PCR
*prmpA2* Rev	GGATGCCCTCCTCCTG		
*iucA* Forw	AATCAATGGCTATTCCCGCTG	239	PCR
*iucA* Rev	CGCTTCACTTCTTTCACTGACAGG		
REP Forw	NNNNCGNCGNCATCNGGC	–	REP-PCR
REP Rev	NCGNCTTATCNGGCCTAC		
ERIC Forw	ATGTAAGCTCCTGGGGATTCAC	–	REP-PCR
ERIC Rev	AAGTAAGTGACTGGGGTGAGCG		

### Purification of OMVs

hvKp was incubated in Luria–bertani (LB) medium (Hope Bio-Technology Co., China) at 37°C, for the preparation of OMVs [26]. To remove dead cells, foreign proteins, and vesicles larger than 1 µm from the bacterial solution, differential centrifugations (5,000 g, 15 min, 4°C; 10,000 g, 30 min, 4°C) were used. The collected supernatant was then filtered through a 0.22-µM membrane filter (Merck Millipore, Germany). After ultracentrifugation at 100,000 g of the residual supernatant at 4°C for 70 min in an SW32Ti rotor (Beckman Coulter, USA), the pellet was resuspended in 500 µL PBS (Gibco Company, USA). OptiPrep (60% iodixanol; Axis-Shield, Norway) density gradient solutions with 5%, 10%, 20%, and 30% density gradients were prepared and distributed into discrete 5%-30% density gradient layers. A 500μL of resuspended OMVs sample from the previous step was deposited on top of the density gradient and centrifuged at 100,000 g for 18 h at 4°C with a SW40 Ti rotor. After centrifugation, the OMVs layer was resuspended in phosphate-buffered saline (PBS). The OMVs suspension was then filtered again through a 0.22-µM membrane filter and cultured on blood agar plates to ensure procedures were sterile. (Merck Millipore, Germany). OMVs were treated with 1 U/μL DNase I (Invitrogen, USA) according to the manufacturer’s protocols and stored at −80°C for further analysis.

### Transmission electron microscope (TEM)

Purified OMVs were visualized by a transmission electron microscope (H-7650, Hitachi, Ltd., Japan). A 20 µL of OMVs was dropwise added into the copper mesh and then stained with 2% uranyl acetate for 5 min. The morphology of OMVs was observed under TEM, operating at 80 kV.

### Nanoparticle Tracking analysis (NTA)

The size distribution of OMVs was determined by NanoSight NS300 instruments (Malvern, UK). OMVs were suspended in sterilized PBS and measured within the optimal dilution. Samples were then injected into the measuring chamber and analyzed by NTA software.

### Quantification of OMVs

BCA Protein Assay Kit (Beyotime Biotechnology, China) was used to determine the protein concentration of OMVs samples according to the manufacturer’s protocols. Simultaneously, the total proteins from OMVs samples were extracted and analyzed using the LC–tandem mass spectrometry (LC-MS/MS) analysis. The peptides were extracted from each sample by a gradient elution at a nanoliter flow rate using the Easy nLC 1200 system (Thermo Scientific, USA). The peptide fragments were separated and analyzed using a Q-Exactive HF-X mass spectrometer (Thermo Scientific, USA) for data-dependent acquisition (DDA) mass spectrometry for 120 min. The mass-spectrometric data were analyzed via MaxQuant 1.6.1.0. Protein database used: uniprot-*Klebsiella pneumoniae* [573]-397877-202102. fasta.

Intravesicular DNA was quantified using the method, as previously described [21]. 50μg of OMVs was treated with 1 U/μL DNase I (Invitrogen, USA) and 50 mg/L proteinase K (Beyotime Biotechnology, China) according to the manufacturer’s protocols. Following DNase and proteinase K treatment, OMVs were lysed for 30 min at 37°C with 0.125% Triton X-100 solution (Sigma-Aldrich, USA). The DNA concentration and purity were then quantified using a Nanodrop (Thermo Scientific, USA); a minimum requirement is that the ratio of OD260/280 ≥ 1.8. Purified DNA was used for subsequent PCR analyses.

### OMVs-mediated transformation

OMVs-mediated transformation experiments were conducted, as previously described [22]; an ESBL-producing *K. pneumoniae* ATCC 700603, which was used as a recipient strain, was incubated in LB broth at 37°C under orbital shaking (150 rpm) for 6 h until the culture reached an OD600 of 0.4. Then recipient strains were resuspended in cold LB broth and adjusted to a concentration of 10^7^ CFU/mL. Purified OMVs were isolated from the donor strains. Following that, 100 µg of hvKp-OMVs was mixed with 200 µl of recipient cells statically at 37°C for 4 h and then for another 4 h under shaking (160 rpm) at 37°C. Fresh LB-broth was added to the mixture, and the culture was incubated overnight under orbital shaking (160 rpm) at 37°C. The collected suspensions were carefully inoculated onto LB agar plates. The successful transformation was preliminary confirmed using the string test. PCR assay was used to determine the presence of *prmpA* and *iroB* as key molecular markers in successful transformants.

### Mucoviscosity assay

A sedimentation assay [27] was used to determine the mucoviscosity of hvKp, cKp-TC, and cKp. In short, cultures incubated at 37°C overnight in LB broth were subcultured in a fresh medium to an OD_600_ of 0.2. The cultures were normalized to OD of 1.0 ml^−1^ after 6 h and centrifuged at 1,000 g for 5 min. The supernatant was gently removed without disturbing the pellet to determine the OD_600_. Stretching bacterial colonies on sheep blood agar plates with an inoculum ring were used to perform a string test.

### Extraction and quantification of capsule

Uronic acid was extracted and quantified in the same way previously described [28]. A 500 µl of bacterial cultures grown for 6 h was combined with 100 µl of 1% Zwitterion 3–14 detergent in 100 mM citric acid (pH 2.0) and incubated at 50°C for 20 min. The cells were then centrifuged, and 250 µl of the supernatant was added to 1.2 ml of 100% ethanol, incubated for 20 min at 4°C, then centrifuged for 5 min at maximum speed. Dried and resuspended the pellet in 200 µl distilled water, then 1.2 ml of 12.5 mM sodium tetraborate sulfate was added, incubated for 5 min at 100°C, and cooled for 10 min. Then, 20 µl of 0.15% 3-phenylphenol in 0.5% NaOH was added. The absorbance at 520 nm after 5 min of incubation at room temperature was measured. The glucuronic acid content, expressed as micrograms OD unit ^−1^, was determined using the standard curve of glucuronate lactone.

### Mouse infection model

The potential toxicity of several *Klebsiella pneumoniae* strains was tested using the mouse bacteremia model [29,30]. Female C57BL/6 mice (7 weeks old) were acquired from Guangzhou Yancheng Biotechnology Service Company and acclimatized for 7 days. The mice were allowed to eat and drink water during the investigation. Simultaneously, the mice were divided into five groups at random (*n* = 5 in each group). Mice in each group were inoculated with 5.0 × 10^5^ colony-forming units (CFU) /mouse of each tested bacterial strain intravenously. Phosphate-buffered saline (PBS) was used as the negative control. Symptoms and mortality rates of the test mice were examined and reported for 120 h after infection. To ensure data consistency, animal experiments were repeated at least twice. Log-rank tests (Mantel–Cox) were used to evaluate survival curves.

### Antimicrobial susceptibility test

The antimicrobial susceptibility of the donor, transformants and rerecipient was conducted using the broth microdilution method according to the Clinical and Laboratory Standards Institute (CLSI) M07 [31].

### REP-PCR

REP-PCR was used to validate that the recipient strains had received the plasmid. Table 1 lists the primers used to perform REP-PCR (repetitive extragenic palindromic sequence-based polymerase chain reaction). The process was performed as described previously [22]. When two strains had the same pattern of bands, they were believed to belong to the same genotype, and a maximum difference between the two bands was acceptable.

## Author contributions

YH, JW, XH, LZ, and QW: conceived the study, supervised the whole project and wrote the manuscript. YH and JW conceptualized the work, performed most of the experiments and drafted the manuscript. MH participated in study design, strains collection and transformation assay. YYH and RZ did the whole-genome sequencing . FB and BY did the mouse infection experiments and participated in data interpretation. JC and XL participated in mucoviscosity assays.

## Supplementary Material

Supplemental Material

## Statistical analyses

Prism 8.0 (GraphPad Software) was used for statistical analysis. A *P* value ≤ 0.05 was used to determine significance. Each data point was repeated at least three times independently.
