# Supplementary material for: Outer membrane vesicles-transmitted virulence genes mediate the emergence of new antimicrobial-resistant hypervirulent Klebsiella pneumoniae
Source: Emerg Microbes Infect. 2022 May 23;11(1):1281–92. doi: 10.1080/22221751.2022.2065935 (PMC9132476; doi:10.1080/22221751.2022.2065935)
Supplement: Supplemental Material [file TEMI_A_2065935_SM7930.pdf]

Supplementary Table 1: The plasmids of hypervirulent *Klebsiella pneumoniae* in the study

name	length	cov	circ	repeat	mult	graph path
contig_6	3027862	41	N	N	1	-9,-9,-9,-9,-8,-2,6,2,8,9
contig_4	721268	50	N	N	1	-9,-8,-2,4,-10
contig_12	526279	53	N	N	1	10,12,-10
contig_5	358517	54	N	N	1	-9,-9,-8,-2,5,-10
contig_14	355144	52	N	N	1	-9,-9,-9,-9,-8,-2,14,-10
contig_13	177011	30	Y	N	1	13
contig_17	113492	22	Y	N	1	17
contig_3	97917	49	N	N	1	-9,-9,-11,-2,3,-10
contig_1	92282	49	N	N	1	10,1,2,8,9,9,9,9,10
contig_7	42894	52	N	N	1	-8,-2,7,-10
contig_16	10455	268	Y	Y	6	16
contig_15	9319	483	Y	Y	10	15

Supplementary Fig 1

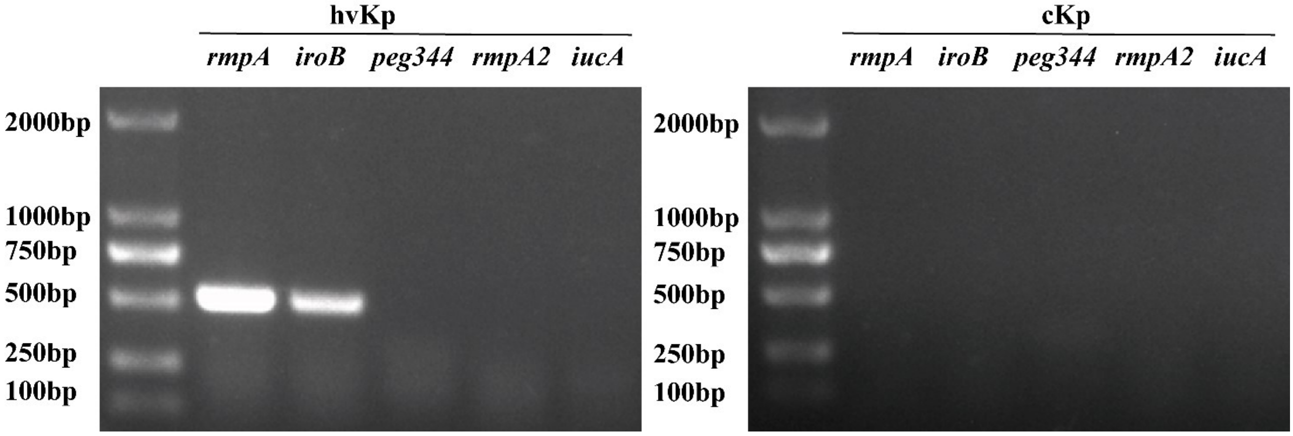

Supplementary Figure 1. PCR amplified fragments of the hvKp and cKp strains in the study.

Supplementary Fig 2

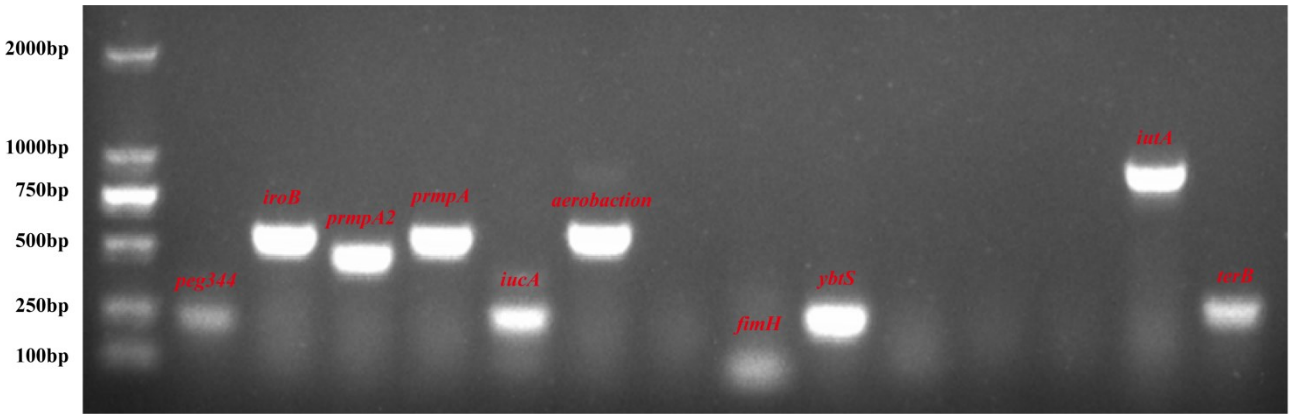

Supplementary Figure 2. PCR amplified fragments of a new hypervirulent *Klebsiella pneumoniae* clinical isolates .

Supplementary Fig 3

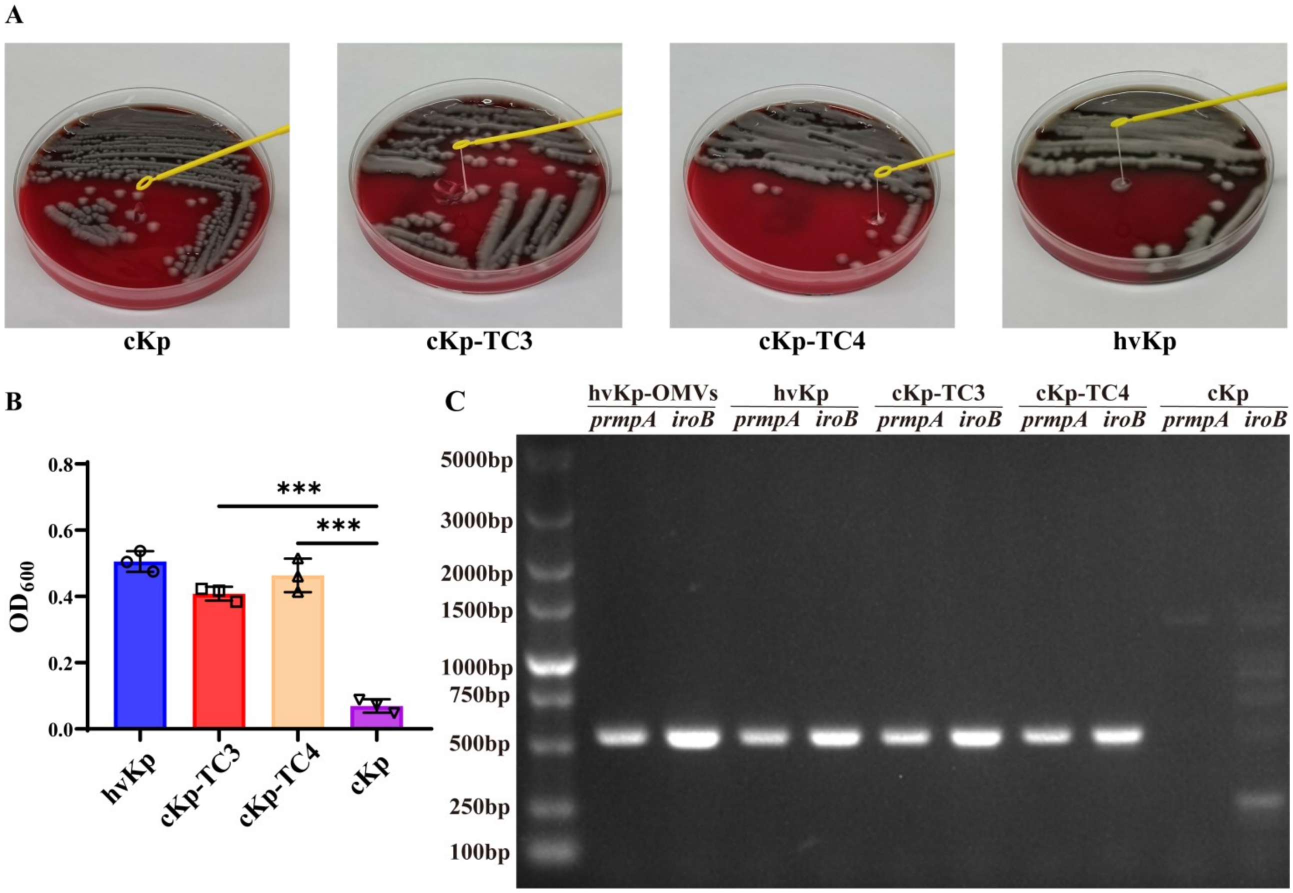

Supplementary Figure 3. OMVs derived from a new hypervirulent *Klebsiella pneumoniae* clinical isolates enhanced level of mucoviscosity and capsule production of cKp strains. (A) String tests on blood agar plates of cKp, cKp-TC3, cKp-TC4, and hvKp strains. (B) Mucoviscosity of hvKp-OMVs,hvKp, cKp-TC3, cKp-TC4 and cKp strains (+$OMVs_{hvKp}$-100μg). Results are presented as mean±SEM, ***P < 0.001.
